# Role of TNFα in pulmonary pathophysiology

**DOI:** 10.1186/1465-9921-7-125

**Published:** 2006-10-11

**Authors:** Srirupa Mukhopadhyay, John R Hoidal, Tapan K Mukherjee

**Affiliations:** 1 Pulmonary Division, Department of Internal Medicine, University of Utah Health Science Center, Salt Lake City, Utah: 84132-4701, USA

## Abstract

Tumor necrosis factor alpha (TNFα) is the most widely studied pleiotropic cytokine of the TNF superfamily. In pathophysiological conditions, generation of TNFα at high levels leads to the development of inflammatory responses that are hallmarks of many diseases. Of the various pulmonary diseases, TNFα is implicated in asthma, chronic bronchitis (CB), chronic obstructive pulmonary disease (COPD), acute lung injury (ALI) and acute respiratory distress syndrome (ARDS). In addition to its underlying role in the inflammatory events, there is increasing evidence for involvement of TNFα in the cytotoxicity. Thus, pharmacological agents that can either suppress the production of TNFα or block its biological actions may have potential therapeutic value against a wide variety of diseases. Despite some immunological side effects, anti-TNFα therapeutic strategies represent an important breakthrough in the treatment of inflammatory diseases and may have a role in pulmonary diseases characterized by inflammation and cell death.

## Background

TNFα is the most widely studied cytokine member of TNF super family. It is secreted by lipopolysaccharide stimulated macrophages and causes necrosis of tumor in vivo when injected into tumor bearing mice [1] and hence bearing the name tumor necrosis factor (TNF). Experimentally, TNFα causes cytolysis or cytostasis of certain transformed cells [2] being synergistic with gamma interferon in its cytotoxicity [3].

TNFα is produced by many different cell types. The main sources in vivo are stimulated monocytes, fibroblasts, and endothelial cells. Macrophages, T-cells, B-lymphocytes, granulocytes, smooth muscle cells, eosinophils, chondrocytes, osteoblasts, mast cells, glial cells, and keratinocytes also produce TNFα after stimulation. Glioblastoma cells constitutively produce TNFα and the factor can be detected also in the cerebrospinal fluid. Human milk also contains TNFα.

Physiological stimuli for the synthesis of TNFα are IL-1, bacterial endotoxins, TNF, platelet derived growth factor (PDGF), and Oncostatin M. In fibroblasts the synthesis of TNFα is stimulated by IFNβ, TNFα, PDGF, and viral infections. In thymic stromal cells the synthesis of TNFα can be induced by neuronal growth factor (NGF). TNFα can also stimulate or inhibits its own synthesis, depending upon the cell type. In epithelial, endothelial, and fibroblastic cells secretion of TNFα is induced by IL-17.

TNFα is a protein of 185 amino acids glycosylated at positions 73 and 172. It is synthesized as a precursor (inactive) protein of 212 amino acids. TNFα converting enzyme (TACE) mediates the cleavage of a membrane associated form of TNFα to inducing the formation of the bioactive soluble TNFα [4]. The secreted protein exists as a multimer of two, three or five noncovalently linked units, but shows a single 17-kDa band in SDS-PAGE under nonreducing conditions [5]. Monocytes express at least five different molecular forms of TNFα with molecular masses of 21.5–28 kDa. They mainly differ by post-translational alterations such as glycosylation and phosphorylation. TNFα is closely related to the 25-kDa protein of TNFβ (lymphotoxin) with around 30% amino acid sequence homology and sharing the same receptors and cellular actions [6]. TNFα mediated signaling plays an important role both in homeostasis and pathophysiology.

## Role of TNFα in physiology and pathophysiology

Over the years it has become increasingly clear that TNFα signaling is a complex series of biological event that involves by at least 29 different tumor necrosis factor receptor (TNFR) family members [7,8]. Under physiological homeostatic conditions the biological functions of this family of cytokines encompasses beneficial and protective effects in both the innate immunity and haematopoiesis, and has a crucial role in organogenesis [7,8]. Members of the TNF super-family are also involved in signaling mechanisms of cellular proliferation, survival and apoptosis.

In vivo, administration of bacterial lypopolysaccharide (LPS) induces high level of TNFα production in animal models and reproduces many common features of septic shock with severe pro-inflammatory reactions [9]. Furthermore, lethal septic shock does not occur in TNFα-deficient mice indicating an important contributory role of TNFα in this syndrome. A high level of TNFα is also observed in human subjects administered bacterial endotoxin [10]. These in vitro and in vivo studies indicate that high level generation of TNFα leads to the exacerbation of inflammatory and prooxidative responses that are important in the pathogenesis of many diseases, including various pulmonary disorders. Due to proinflammatory and prooxidative actions, TNFα complicates many diseases, the most important of which are atherosclerosis [11], rheumatoid arthritis [12], psoriasis [13], inflammatory bowel disease [14], Alzheimer's disease [15] and various pulmonary disorders. This review very precisely describes the roles of TNFα in various pulmonary diseases.

## Mechanism of action of TNFα in pulmonary pathological consequences

Inflammation is believed to be the key event of TNFα-dependent pathophysiological events. Deregulated recruitment of leukocytes and lymphocytes at the inflamed foci leads to injury. TNFα also depletes cellular glutathione (GSH), a cellular antioxidant [16]. Over-expression of TNFα in transgenic mice induces differential changes in redox status and glutathione-regulating enzymes [17] by depleting the total cellular glutathione levels. In vitro studies also demonstrate that TNFα depletes cellular GSH levels. Treatment of human pulmonary artery endothelial cells with TNFα decrease the GSH levels of the cells [18] and this depletion of glutathione enhances the endothelial cell susceptibility to oxygen toxicity [19]. The exact mechanism through which TNFα decreases the levels of glutathione in pulmonary tissues has not yet been fully determined.

Both in vitro and in vivo studies show that TNFα stimulates the reactive oxygen species (ROS) generation from pulmonary and non-pulmonary tissues. Upon TNFα stimulation a high level generation of ROS is observed in human endothelial cells [20], and neutrophils [21]. Of the different cell types, ROS derived from neutrophils takes a very important role in TNFα-dependent alteration of pulmonary vasoreactivity [21]. However, another study observing the mechanism of adherence of neutrophil on human pulmonary microvascular endothelial cells show endothelial cells are the source of TNFα stimulated ROS not neutrophil [22]. Over the years it is generally assumed that in physiological homeostatic conditions most of the ROS generation takes place at the phagocytic cells such as macrophages or neutrophils and the ROS generation by nonphagocytic cells is a minor fraction to that of the phagocytic cells.

At the subcellular level NADPH oxidase and mitochondria are the potential sites of TNFα-dependent ROS generation and subsequent oxidative stress in endothelial cells [23]. Recent studies indicate that NADPH oxidase and mitochondria are linked through a feedback mechanism [23]. Interestingly, a recent study has reported that the two sources ROS (mitochondria and NADPH oxidase) may have divergent pathways in endothelial cells: The mitochondrial pathway is suppressed by rotenone and appears to be directly involved in TNFα induced apoptosis by activating caspase 3. Another pathway is a membrane-dependent pathway that is associated with the NADPH oxidase and protects against TNFα-induced cell death by activation of small GTPase Rac1 (a component of NADPH oxidase) [24]. Thus, depending upon the type of stimulus and the levels of generation of ROS, cells may undergo either pro-survival or pro-apoptotic pathway in response to TNFα.

**Figure 1:**
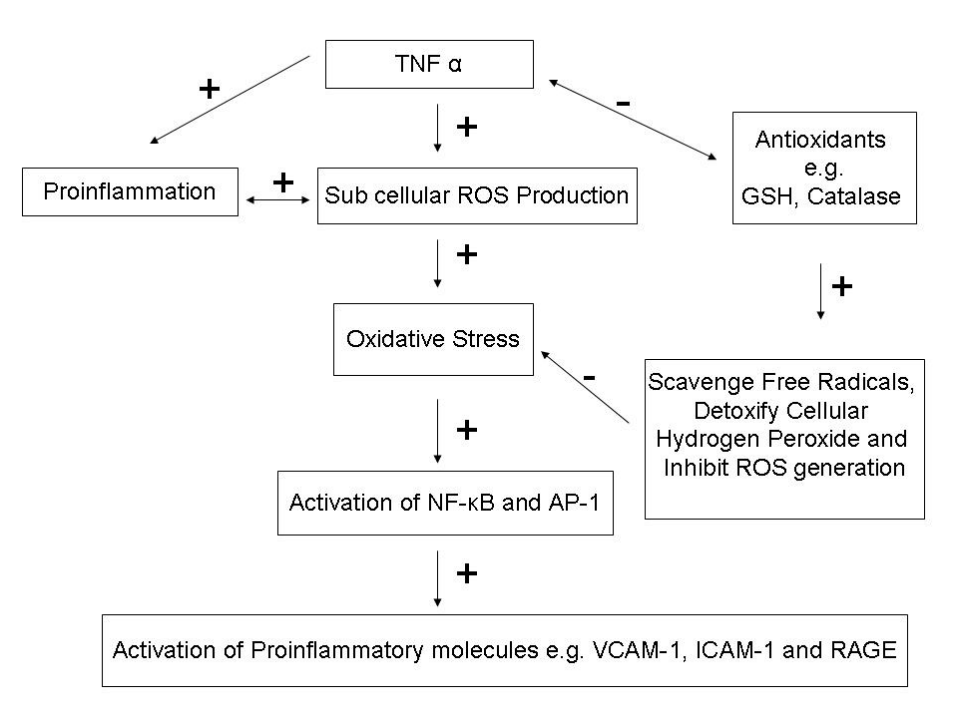
Schematic diagram of the mechanism of action of TNFα. While TNFα-dependent activation of reactive oxygen species (ROS) generation enhances oxidative stress of cells and subsequent activation of pro-inflammatory and pro-oxidative transcription factors nuclear factor kappa-B (NF-κB) and the activator protein one (AP-1), antioxidants namely GSH attenuates oxidative stress and subsequent activation of NF-κB and AP-1. NF-κB and AP-1 are involved in the activation of pro-inflammatory molecules like, vascular cell adhesion molecule one (VCAM-1), intercellular adhesion molecule one (ICAM-1) and receptor for advanced glycation end products (RAGE). + Indicates activation, - indicates inhibition → and ↔ indicate one way and two way flow of signals respectively.

During the course of ROS generation various adhesive and proinflammatory molecules are generated that are responsible for the complications of various inflammatory disorders [25] (Fig. 1). In short, induction of cellular inflammatory reactions, enhancement of oxidative stress and increased expression of various proinflammatory molecules altogether create the basic foundation of biological action of TNFα.

## Role of TNFα in pulmonary pathophysiology

**Figure 2:**
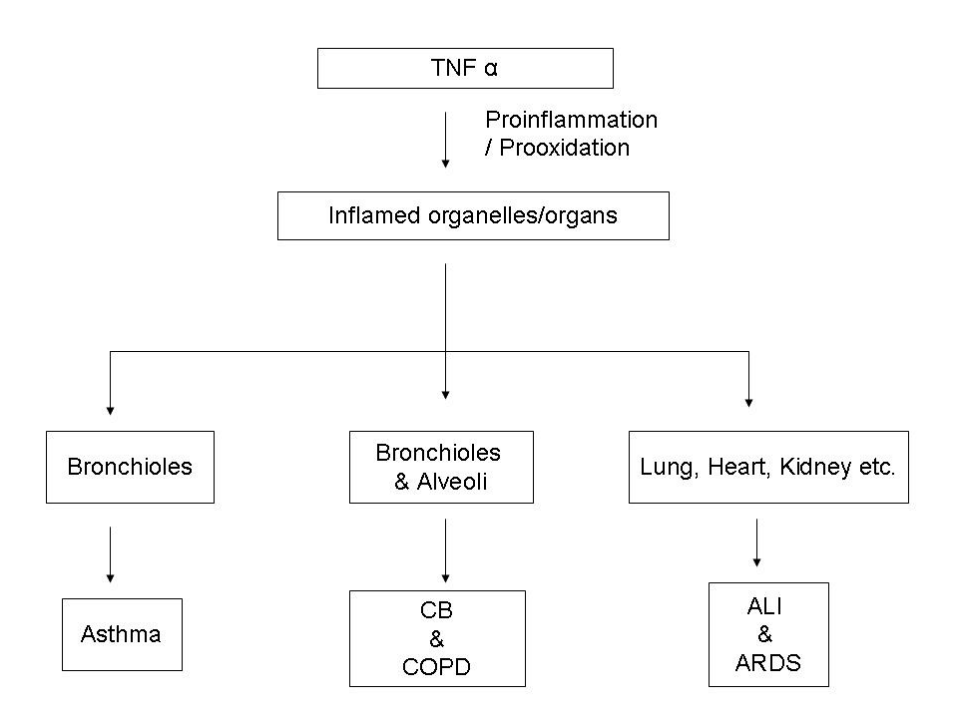
Schematic diagram of the effects of TNFα on various pulmonary tissues. Asthma is mainly a disease of bronchioles; Chronic Bronchitis (CB) and chronic obstructive pulmonary disease (COPD) are the disease of bronchioles and alveoli. In acute lung injury (ALI) and acute respiratory distress syndrome all the vital organs of the body namely lung, heart, kidney, liver etc are affected.

TNFα plays a significant role in many inflammatory diseases of lung. The most important lung diseases affected by TNFα include chronic bronchitis (CB), chronic obstructive pulmonary disease (COPD), asthma, acute lung injury (ALI) and its severe form acute respiratory distress syndrome (ARDS) (Fig. 2). The diverse functions of TNFα significantly depend upon the duration and quantity of TNFα expression. In addition, genetic background and timing of TNFα expression and release also determine its function and its diversity of the immune response. Thus, high level generation of TNFα is linked to the pathophysiological consequences in a number of pulmonary diseases. The following section discusses the role of TNFα in various pulmonary diseases.

## Role of TNF-α in asthma

Asthma is an inflammatory disease, characterized by airway hyperreactivity, chronic eosinophilic inflammation, episodes of reversible bronchoconstriction, and mucus hypersecretion [26]. The eosinophilic inflammation associated with asthma is typically coupled with increased numbers of CD4^+ ^T lymphocytes that produce increased levels of TH_2_type cytokines and decreased levels of γ-interferon [27]. Of note, different allergic phenotypes cannot be distinguished strictly on the basis of TH_1 _and TH_2 _cytokines [28]. Although murine immune system differ than human immune system, in general asthma and allergic disorders are casually characterized by elevated Th_2 _cytokines (IL-4, IL-5, IL-13) and the chronic inflammatory response in asthmatic airways is maintained by Th_1 _cytokines [28].

Several lines of evidences indicate that high levels of TNFα are directly linked to asthmatic complications. Most of the data are based on experimental observations and in-vitro studies and a clear understanding of the exact role of TNFα in childhood or adult asthmatic patients is yet to be determined. In bronchi epithelial, endothelial and smooth muscle cells are the primary targets of TNFα. It causes substantial damage in the normal bronchial epithelial cells [29] and the bronchi of the allergic mouse model [30]. In severe bronchial allergic inflammation TNFα-dependent leakage of epithelial and endothelial cells may have severe pathophysiological consequences [30]. In bronchial smooth muscle cells TNFα-dependent hyperplasia and vasoconstriction are important. Severe, persistent asthma is characterized by airway smooth muscle hyperplasia, infiltration of inflammatory cell into smooth muscle and increased expression of an array of cytokines including IL-4, IL-13, IL-1β and TNFα. TNFα has the potential to alter the expression of cell surface receptors such as CD40 and OX40 present on the airway smooth muscle cell [31]. In allergic asthma in the presence of low antigen concentrations TNFα is particularly involved in the potentiation of histamine release [32]. TNFα causes vasoconstriction by secondary release of endothelin 1 [33]. In the murine asthmatic model, the late-phase airway hyperresponsiveness and airway inflammation is mediated by TNFα-dependent activation of phospholipase A2 [34]. It is further suggested that the augmentation of the acetylcholine-induced contractile response evoked by TNFα might be mediated by an upregulation of small G proteins i.e. RhoA in rat bronchial smooth muscle cells [35]. The exact role of Rho family members in controlling the TNFα-dependent signaling in human pulmonary tissues is yet to be determined.

## Role of cytokines other than TNFα in asthma

Recent studies using TNFα knockout mice or anti-TNFα antibodies have demonstrated that elimination of TNFα bioactivity alone is not sufficient to abrogate the murine inflammatory response to aerosolized ovalbumin [36]. In allergic inflammation, other proinflammatory cytokines appear to act together with TNFα after repeated antigenic challenges. Increased levels of IL-2, IL-4 and IL-5 are detected in the lung homogenates from ova-sensitized and challenged TNFR (+/+) and TNFR (-/-) mice compared with those from control mice. Mice deficient in TNFRs may have an altered immunological feedback mechanism(s), resulting in an accentuated TH_2_-type immune response in ova-induced allergic inflammation. Numerous studies have suggested that IL-4 and IL-5 are important in the pathogenesis of allergic inflammation [37]. Another recent study examined the effects of antibody neutralization of IFNγ, IL-4 or TNFα in ova-sensitized mice challenged for 8 consecutive days with aerosolized ova. The experimental data showed that only neutralization of IFNγ attenuated airway hyperreactivity and eosinophil influx into the bronchoalveolar lavage (BAL) fluid [38]. Recent clinical trials on human have shown that sequestration of IL-5 by specific antibodies led to reduced IL-5 levels and eosinophil numbers in blood and sputum, but had no effect on lung function or bronchial hyper-responsiveness [39] or had a minor effect [40]. Studies with IL-4 neutralization by soluble IL-4 receptor were also showing reduced asthma symptoms and improved lung function [41]. Taken together, these data suggest that cytokines other than TNFα may also have important roles in controlling asthma symptoms.

## Role of TNFα in chronic obstructive pulmonary disease and chronic bronchitis

Chronic obstructive pulmonary disease (COPD) is characterized by chronic inflammation in the airway lumen along with increased numbers of neutrophils, macrophages, CD8^+ ^T cells or mast cells in the airway walls and alveolar compartments [42,43]. This complex disease state consisting of emphysema (centrilobular and panacinar), small airway disease and chronic bronchitis (CB) with air flow obstruction [44]. The cause for CB as such may be diverse, including infections and air pollution, but not necessarily smoking. CB without airflow obstruction does not need to result in emphysema. However, smoking is the most significant risk factor for patients with CB plus airflow obstruction/COPD, although an important (though small) subset of CB and COPD patients are nonsmokers. However given the fact that only 15%–20% of smokers develop CB or COPD; which largely indicates that apart from smoking a genetic component is probably an operating factor for COPD [45,46]. In addition, patients with COPD/CB often do not respond to corticosteroid therapy, whereas patients with CB alone do [46]. Of note, very recently, a comprehensive review article discussed in details the differences of CB alone and COPD [44].

Experimental animal models show that TNFα over-expression induces the pathological changes similar to emphysema and pulmonary fibrosis. For example, in mice airspace enlargement, loss of small airspaces, increased collagen, thickened pleural septa and increased chest and lung cavity volume are some of the changes mediated by TNFα over-expression [47]. Additionally, TNFα and TNFβ genes contain several polymorphisms including a transition at -308 region in TNFα gene (promoter) where guanine (G) is replaced by adenine (A). Similarly, in the first intron of the TNFβ gene at 252 region, A is replaced by G. These single nucleotide polymorphisms (SNPs) have been associated with COPD. For example, an association between the TNFα -308A allele and COPD was recently found in a Taiwanese population. The patients with chronic bronchitis and impaired lung function had a prevalence of the TNFα -308A allele as compared to the control subjects [48]. In contrast to these observations it was shown from a group in Thailand that TNFα gene promoter polymorphisms are not associated with smoking-related COPD [49]. Moreover, another study also revealed that SNPs are independent factors in COPD for the Han population in Beijing [50]. These results indicates a race dependent differences exists in various Asian population and TNFα gene promoter polymorphisms may not be very important in the development of COPD at least in these population. In this regard a recent study indicates that subjects of COPD express different patterns of proinflammatory mediators in bronchial secretions, which appears to be modulated according to the etiological cause. In particular, TNFα concentration per se enables the recognition of COPD exacerbations due to *Pseudomonas Aeruginosa *infection, while IL8 + IL1β levels prove helpful in discriminating common bacterial infection from viral infections and noninfectious causes [51]. Therefore, the concentration of TNFα is likely to depend on the type of infections in COPD patients [51].

## TNFα in acute lung injury and acute respiratory distress syndrome

Acute lung injury (ALI) is characterized by noncardiogenic edema, pulmonary inflammation and severe systemic hypoxemia. Acute respiratory distress syndrome (ARDS) is the severe form of ALI. Systemic sepsis and pneumonia are common predisposing factors for ARDS, which can serve as the initial manifestation of multisystem organ failure [52]. One of the earliest manifestations of ALI is the activation of antigen presenting cells like macrophages (alveolar and interstitial), upregulation of cell surface adhesion molecules and subsequent production of cytokines and chemokines that induce sequestration of neutrophils within the pulmonary microvasculature. These cells migrate across the endothelium and epithelium into the alveolar space and release a variety of cytotoxic and proinflammatory compounds, including proteolytic enzymes, ROS and reactive nitrogen species (RNS), cationic proteins, lipid mediators and additional inflammatory cytokines [53]. This perpetuates a vicious cycle by recruiting additional inflammatory cells which in turn produce more cytotoxic mediators, leading to the profound injury of the alveoli-capillary membrane and respiratory failure.

Both TNFα and TNFβ subtypes appear in the circulation during the onset of sepsis-induced lung injury, implicating these cytokines as potential inflammatory mediators of this disease [54]. On the basis of several experimental studies, TNFα along with other cytokines is suggested as important early mediators of ALI [55]. Despite these findings TNFα level present in the plasma, bronchoalveolar lavage (BAL) and pulmonary edema fluid have not been consistently correlated with clinical outcomes in patients at risk or already diagnosed with ALI [56]. Interestingly, this study did not consider the interplay between TNFα and the two cell surface receptors that mediate its inflammatory effects: TNF-R_I _and TNF-R_2_. A number of studies identified TNF-R_1 _in pulmonary tissues [57]. Recent studies demonstrated that a mixture of TNFα, IL-1β and IFNγ, which are all known to be present in the lungs of the patients with ARDS, stimulated the release of soluble TNF-R_1 _but not TNF-R_2 _from cell surfaces. These soluble receptors (sTNFR) of TNFα bind to circulating TNFα and compete with cell surface receptors for TNFα binding [58,59]. Thus, the lack of correlation between TNFα levels and clinical outcomes may be attributed to sTNFR-TNFα complex formation that decreases the bio-availability and binding of TNFα into the membrane associated TNFR.

Interestingly, an association has been found between increased risk of ARDS and the polymorphisms of surfactant protein B (SP-B) present in lung [60]. SP-B is essential for the maintenance of biophysical properties and the physiological function of the pulmonary surfactant. Pulmonary surfactant is a lipid-protein complex that lowers surface tension along the alveolar epithelium, thereby promoting alveolar stability and preventing collapse of alveoli during ventilation [61]. Destruction of surfactant in lung results in an increase in the surface tension at the air-liquid interface, which results in alveolar and peripheral airway collapse and potential injury [62]. Interestingly, TNFα inhibits the SP-B promoter activity in a human lung adenocarcinoma cell line NCI-H441 [63]. TNFα also down regulates surfactant protein A (SP-A) gene expression in lung epithelial cells via the p38 MAPK signal transduction pathway [64]. Therefore, loss of the lung surfactant during severe septic conditions may be related to increased TNFα activity.

## Anti-TNFα therapy as a way to control pulmonary diseases

Involvement of TNFα in various inflammatory disorders has led to the use of pharmacological agents that can either suppress the production of TNFα or block its action. A variety of candidates are being studied including inhibitors of TNFα mRNA transcription (e.g. pentoxifylline and phosphodiesterase inhibitors) [65,66], accelerators of TNFα mRNA degradation (e.g. thalidomide) [67], inhibitors of TNFα protein translation (e.g. tetravalent guanylhydrazones) [68] and the metalloproteinase inhibitors that prevent the cleavage of the 26 kDa membrane-bound protein to the active 17 kDa molecule [69]. Other approaches include TNF receptor fusion proteins [70] and monoclonal antibodies raised against TNFα. The latter have been used in human subjects who have rheumatoid arthritis, usually as a humanized murine antibody [71]. Therefore, these pharmacological agents may have potential therapeutic value for a wide variety of TNFα-mediated disorders.

Anti-TNFα therapy for various pulmonary diseases overall has shown various levels of success. For example, anti-TNFα therapy prevents ventilation induced lung injury in rats [72]. In another experimental rat model anti-TNFα therapy ameliorates ozone-induced lung injury [73]. Recent studies also identified TNFα as a novel therapeutic target in symptomatic corticosteroid dependent asthmatic patients [74]. Finally, another case study illustrates a successful treatment of bronchiolitis obliterans in a bone marrow transplant patient with TNFα blockade [75]. However, the effects of anti-TNFα treatment presently require further confirmation in controlled trials.

In contrast to the experimental studies several recent clinical trials showed either very little or no beneficial effect during anti-TNFα therapy in pulmonary diseases. For example, double blind, placebo controlled randomized, phase two trial of infliximab for a short term on 22 current smokers with mild to moderate COPD showed no beneficial effects [76]. However, as pointed out in several reviews [77,78] infliximab is just one of the many antagonists of TNFα and its receptors and its effect may need longer treatment instead 6 weeks of treatment. Of note, a positive effect has in general been seen in the treatment of rheumatoid arthritis after 12 weeks of treatment. In contrast to this view in a recent meta analysis study it has been shown thatan increased risk of serious infections and a dose-dependent increased risk of malignancies in patients with rheumatoid arthritis treated 12 weeks with two anti-TNF antibodies (infliximab and adalimumab) [79]. Similarly in mild to moderate asthmatic subjects, TNFα antagonism was not be effective for preventing allergen-mediated eosinophilic airway inflammation. Diffused alveolar hemorrhage, delayed hypersensitivity reaction and ARDS syndrome have been also reported after infliximab treatment in Crohn's disease patients [80,81]. These and other similar kinds of studies apparently present a serious challenge for the selective or partial usefulness of anti-TNFα therapy.

## Conclusion

Anti-TNFα therapy demonstrates overall various levels of success in arthritis, psoriasis, inflammatory bowel disease and some other inflammatory disorders. However, no conclusive data has yet been identified to prove the efficacy of anti-TNFα therapy for major pulmonary diseases. Genetic polymorphism of the TNFα promoter and heterogeneity of the TNFα receptor gene may play a significant role in the non-responsiveness of anti-TNFα therapy. The most important adverse effects of anti-TNFα therapy include the alterations of early and delayed type hypersensitivity and dampened cell-mediated immune responses. Of note, TNFα is one of the normal immune molecules of the body. Therefore, future studies leading to a combination of drugs that protect the cellular immunity system but selectively block TNFα action may be more insightful for use to overcome the side effects of anti-TNFα therapy in the long-term. Again, proinflammation and prooxidation is the root cause of the complications of various inflammatory diseases. Since TNFα directly induces the oxidative stress of the cells by depleting the GSH (the most abundant and vital antioxidant of the body) and therefore elevates the ROS levels of the cells, it would be interesting to check the effectiveness of combination of drugs including GSH enhancer and low dose anti-TNFα antibody to effectively combat the side effects of TNFα therapy. Lastly, future studies involving 12 weeks or long term anti-TNFα therapy may be useful against COPD (as observed in rheumatoid arthritis patients) and effectively combat pulmonary pathological consequences.
